# Impact of the hierarchical medical system on the perceived quality of primary care in China: a quasi-experimental study

**DOI:** 10.1186/s41256-024-00398-3

**Published:** 2025-02-19

**Authors:** Yaxin Zhao, Xiaohui Zhai, Zhongliang Zhou, Zixuan Peng, Chi Shen, Xiaojing Fan, Sha Lai, Peter C. Coyte

**Affiliations:** 1 College of Humanities & Social Development, Northwest A&F University, Xianyang, 712100 Shaanxi China; 2 School of Public Health, Health Science Center, Xi’an Jiaotong University, No.76 Yanta West Road, Xi’an, 710061 Shaanxi China; 3 School of Public Policy and Administration, Xi’an Jiaotong University, No.28 Xianning West Road, Xi’an, 710049 Shaanxi China; 4 School of Public Health, Southeast University, Nanjing, 211189 Jiangsu China; 5 Dalla Lana School of Public Health, Institute of Health Policy, Management & Evaluation, University of Toronto, 582-155 College Street, Toronto, ON M5T 3M7 Canada

**Keywords:** Primary care, Patients’ perceived quality, Difference-in-differences, Hierarchical medical system, China

## Abstract

**Background:**

Although the implementation of a hierarchical medical system (HMS) has been shown to improve the allocation of medical resources and patient health-seeking behaviour, its role in patient’s perceived quality of primary care remains unexplored. This study aimed to assess the impact of HMS implementation on rural and urban residents’ perceived quality of primary care.

**Methods:**

Data were obtained from the China Family Panel Study for 2012, 2014, 2016, and 2018. A total of 40,011 rural and 22,482 urban residents were included in the research participants for analysis. This study adopted a quasi-natural experimental design, and the multiple-period difference-in-differences method was used to capture changes in patient’s perceived quality of primary care before and after the introduction of HMS.

**Results:**

We found that HMS implementation declined the perceived quality of primary care by an average of 18% among rural residents (OR: 0.82, 95% CI 0.68–0.99), while there was no significant change among urban residents (OR: 1.13, 95% CI 0.87–1.46). There was a 24% reduction in the perceived quality of primary care (OR: 0.76, 95% CI 0.61–0.96) one year after HMS among rural residents, and there was no statistically significant difference two years after HMS. After HMS implementation, the level of perceived quality of primary care by rural patients with chronic diseases decreased by 72% (OR: 0.28, 95% CI 0.11–0.78).

**Conclusions:**

HMS has a limited effect on improving residents’ perceived quality of primary care, especially for those living in rural areas. Policymakers are suggested to establish a quality monitoring system that incorporates patient experience as an essential standard to systematically evaluate the impacts of the HMS, with more efforts being put into helping vulnerable groups such as residents under 60 years old and patients with chronic diseases.

**Supplementary Information:**

The online version contains supplementary material available at 10.1186/s41256-024-00398-3.

## Introduction

Primary care plays a fundamental and vital role in the management of common diseases, chronic diseases, and infectious diseases [1–3]. High-quality primary care has been shown to yield health benefits, including a reduction in health inequity and healthcare spending [4–6]. As such, assessing and improving the quality of primary care has become a priority for countries around the world. Most previous studies assessed the quality of primary care based on the model from Donabedian’s structure-process-outcome approach theory and used the SERVQUAL 15 questionnaire, which is widely employed [7, 8].

With rapid development in the economy and medical technology, market-oriented and patient-centred care makes patients’ expectations and feelings a basic standard of high-quality primary care [9]. As patients are direct targets of healthcare services, their feelings and experiences are more convincing in assessing the quality of healthcare services. The perceived quality of primary care is patient-centred and emphasizes the true feelings of patients, which is closely related to patient satisfaction [10]. Therefore, patients’ perceived quality of primary care has become an important indicator of concern for medical quality evaluation. Policymakers around the world are increasingly inclined to use patients’ perceived quality of care, such as accessibility, equitability and satisfaction of healthcare providers, rather than actual medical technology quality indicators to evaluate the quality of healthcare services [11–13].

Many studies have verified that healthcare reform can improve patients’ perception of the quality of healthcare services. A Northern California intervention study found that lean primary care redesigns yielded health improvements in terms of enhancing patient experiences of care [14]. André et al. showed that introducing a family medicine group in Quebec improved the perceptions of relational and informational continuity of care [15]. A cross-sectional study in China suggested that family practice services can promote higher perceived quality of primary care [16], but very little research exists on the net impact of health reform in China on primary care quality using longitudinal data at the nationwide level.

As the largest developing country in the world, China has been striving to improve the quality of primary care. The Chinese government introduced the Hierarchical Medical System (HMS) in September 2015 and initiated four categories of measures to enhance the quality of primary healthcare services [17], including the first consultation system, training and education of health workforce in grassroots, technical support of primary care facilities and two-way referral mechanism. Improving the quality of primary care is an urgent requirement for promoting the development of a healthy China and HMS. A better understanding of the unintended consequences of HMS is essential for medical reform to succeed. Therefore, this study aimed to evaluate the impact of HMS on the perceived quality of primary care among rural and urban residents in China using a quasi-experimental design and examine the long-term and marginal impact of HMS.

## Methods

### Identification strategy

The difference-in-differences (DID) design can effectively address endogeneity and is the most widely used method for policy estimation. Owing to the different implementation times of HMS in various provinces in China, a time-varying DID design was adopted (see Supplemental Table 1). The design emphasizes the addition of a control group before and after differences, comparing the differences between the intervention group before and after differences and the control group before and after differences, forming a quasi-experimental design with a control group before and after HMS design [18]. The time-varying DID model was implemented as an interaction term between the time dummy and treatment group (provinces that piloted the HMS were identified as the intervention group) dummy variables in the regression model. The interaction term between the group and the time dummy takes a value of 1, meaning that the province implemented the HMS at a time point before which the value of the interaction term would be equal to 0. We set the policy time to 2017 if the provinces implemented HMS in the second half of 2016 (see Supplemental Table 1).

### Data source

**Fig. 1:**
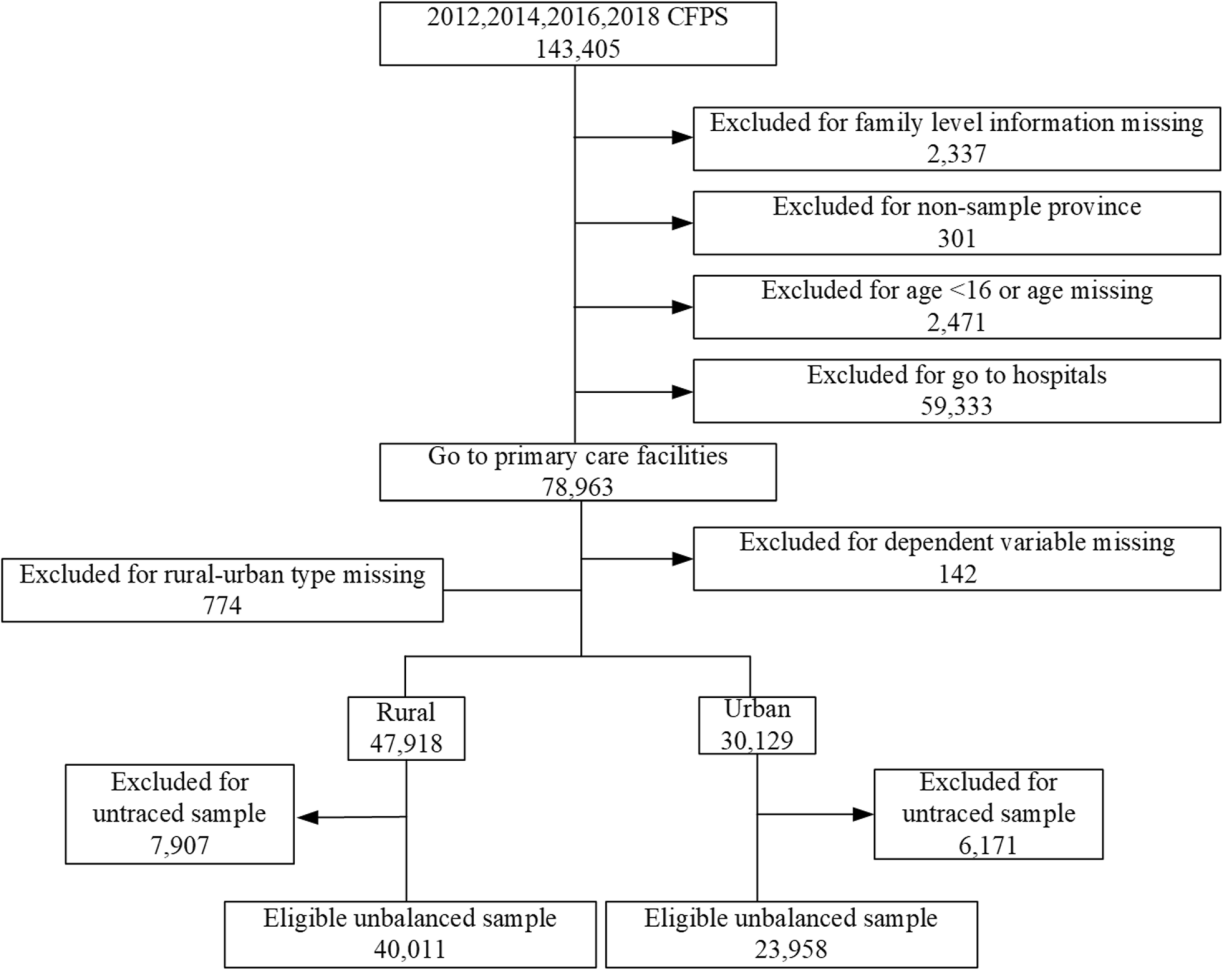
Flow chart of data processing

The individual-level data were derived from the China Family Panel Studies (CFPS) for 2012, 2014, 2016 and 2018. CFPS is a nationally representative survey launched by the Institute of Social Science Survey of Peking University and is conducted every two years. The survey adopts a probability proportional to size sampling method, with its baseline sample covering 25 provinces/cities/autonomous regions [19]. The questionnaire-based face-to-face interviews were conducted to collect information on individuals’ demographics, income, health status, perceived quality of primary care, and so on. According to the aim of our study and the definition of primary care facilities, residents who usually go to primary care facilities if they are sick were included in the analysis. Due to the differences in medical and health systems between urban and rural areas in China, uneven distribution of medical resources, and differences in health awareness among urban and rural residents, this study focused on the region of residents, using the variable (urban vs. rural area) based on the CFPS recommended to assess the short- and long-term impact of HMS on the quality of primary care perceived by rural and urban residents in China. After excluding samples with missing or invalid values, we obtained a longitudinal dataset consisting of 40,011 rural residents and 23,958 urban residents (see Fig. 1 and Supplemental Table 2).

### Measures

Patients’ perceived level of quality of primary care is the outcome variable of interest and an important dimension of quality of care, which has been widely used. Patients’ perceived quality was measured using the question “What do you think about the medical quality of the medical institutions you usually visit?”. Responses were rated on a five-point Likert scale (see Supplemental Tables 3 and 4). Because we only included residents who usually go to primary care facilities, the outcome variable reflects the overall evaluation of the perceived quality of primary care. To interpret the perceived quality of primary care more comprehensively, we also used patients’ satisfaction with the medical conditions of primary care facilities as an alternative measure of the perceived quality of primary care in the robustness analysis.

The interaction term between HMS implementation and time dummies was included as the key independent variable (see Supplemental Table 3). According to Andersen’s behavioural model of healthcare utilization and the existing literature, several variables were incorporated as potential determinants of the perceived quality of primary care [16, 20]. We controlled the predisposing factors of demographic variables (including gender, age, marital status, and education), enabling factors of annual household income per capita, and health-related status (including whether they were covered by basic medical insurance, self-reported health status, and chronic diseases) [21]. According to the existing literature [22], we also controlled the number of health workers in primary care facilities per 1000 residents, which were obtained from the 2013, 2015, 2017 and 2019 China Health and Family Planning Statistical Yearbooks.

### Statistical analysis

We constructed a time-varying DID model and used an ordered logit model to assess the impact of HMS on the perceived quality of primary care. We included time- and individual-level fixed effects in the DID model to control the potential endogeneity problems arising from unobserved confounders [23]. To estimate whether the HMS had a lasting effect, we performed an additional analysis to explore the marginal impact of the HMS on each category of perceived quality of primary care. The ordered logit model is expressed as follows:

1 $$ologit\;Y_{it} = \alpha + \beta did_{\_HMSit} + \lambda X_{it} + \mu_{i} + \gamma_{t} + \varepsilon_{it}$$

where ${ologit Y}_{it}$ represents the level of the quality of primary care perceived by individual $i$ in year $t$; ${{did}_{\_HMS}}_{it}$ is the interaction between the group and time dummies and the $\beta$ denotes the impact of HMS on the perceived quality of primary care; ${X}_{it}$ denotes a vector of control variables; ${\mu }_{i}$ and ${\gamma }_{t}$ denote the individual- and time-level fixed-effects respectively; ${\varepsilon }_{it}$ is the remaining error term.

We constructed a linear two-way fixed-effects model to test the parallel trend assumption. If the coefficient in the pre-intervention period was insignificant, means that the trend of perceived quality of primary care was the same across the pre-intervention period and in accord with the parallel trend hypothesis [24]. All statistical analyses were performed using Stata software, version 14. The results are shown as odds ratios (OR) with 95% confidence intervals (CI).

### Robustness checks

We performed a set of checks to test the robustness of our key findings. First, we constructed a multiple-time-period DID model with an ordinary least squares (OLS) estimator to measure the impact of the HMS. Second, we used patient satisfaction as another measure of the perceived quality of primary care. Third, we performed counterfactual testing and moved the policy implementation time forward to estimate the impact of HMS. In addition, we conducted a subgroup analysis to explore the variations in policy effects between patients with and without chronic diseases.

## Results

### Descriptive summary

The descriptive statistics of the sampled individuals are shown in Table 1. We found that 50.29% and 51.52% of rural and urban residents were female, respectively. The mean ages of the rural and urban samples were 47.82 and 45.96, respectively. Of the respondents, 82.93% were married and 92.73% were covered by basic medical insurance. The per capita household income of the rural and urban residents was 9,200.54 CNY and 14,274.21 CNY respectively. We found that 33.78% of rural and 38.95% of urban residents reported having “good” health status, 13.46% of rural residents, and 12.24% of urban residents were diagnosed with chronic diseases. The number of health workforce per 1,000 population in primary care facilities was 1.61 and 1.66 in rural and urban areas respectively.

**Table 1:** Descriptive characteristics of rural and urban patients

Variables	Description	Rural	Urban
Gender	Female	20,122 (50.29)	12,342 (51.52)
	Male	19,889 (49.71)	11,616 (48.48)
Age (years)	Continuous variable	47.82 (15.81)	45.96 (16.15)
Marital status	Unmarried	3885 (9.71)	2994 (12.5)
	Married	33,181 (82.93)	19,289 (80.51)
	Divorced or widowed	2943 (7.36)	1675 (6.99)
Education status	1 = Illiterate, reference	14,885 (37.35)	5400 (22.65)
	2 = Primary school	10,320 (25.9)	5086 (21.34)
	3 = Junior middle school & above	14,645 (36.75)	13,352 (56.01)
Household income per capita (CNY)	Continuous variable	9200.54 (14,925.94)	14,274.21 (18,513.39)
Basic health insurance	Uninsured	2908 (7.27)	3028 (12.64)
	Insured	37,103 (92.73)	20,930 (87.36)
Self-reported health (SRH)	1 = Poor, reference	7002 (17.5)	2798 (11.68)
	2 = Fair	6324 (15.81)	4296 (17.93)
	3 = Good	13,514 (33.78)	9331 (38.95)
	4 = Very good	7849 (19.62)	4539 (18.95)
	5 = Excellent	5318 (13.29)	2994 (12.5)
Chronic diseases	No chronic diseases	34,619 (86.54)	21,022 (87.76)
	Diagnosed with chronic diseases	5384 (13.46)	2933 (12.24)
Number of health workers in primary care facilities (per 1000 residents)		1.61 (0.24)	1.66 (0.27)

Age, economic level and number of health workforce in primary medical institutions per 1000 residents were performed as Mean (SD). Categorical variables are shown as N (%))

SRH, Self-reported health

^a^means urban employees’ basic medical insurance, urban residents’ basic medical insurance and new rural cooperative medical insurance

### Analysis of common trends in the perceived quality of primary care

**Fig. 2:**
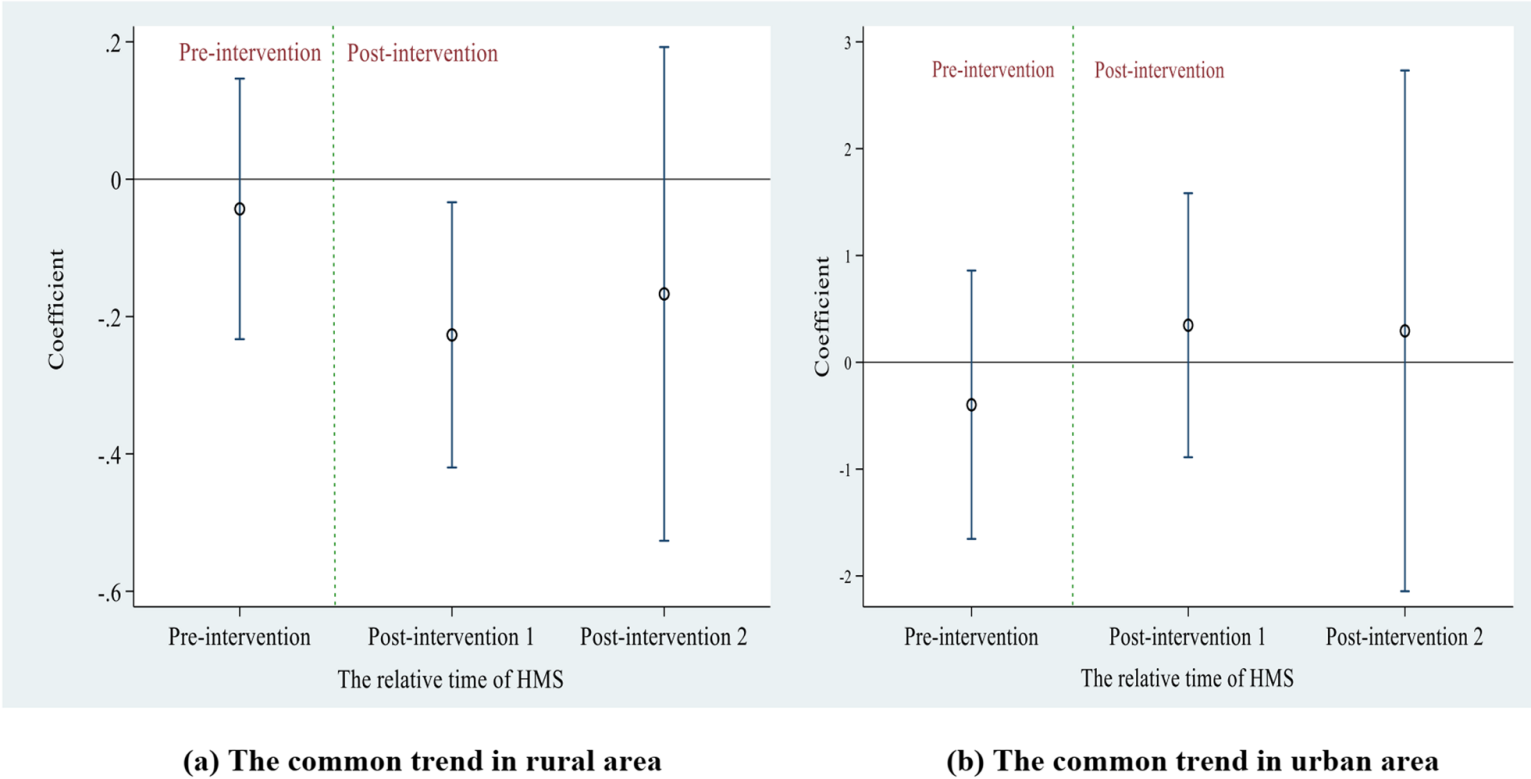
The common trends of the perceived quality of primary care before and after HMS

Figure 2 presents the common trends among rural and urban residents before and after policy implementation. The coefficients were not significant for either rural or urban residents before the implementation of the HMS. There was no significant difference in the perceived quality of primary care in the pre-intervention period among rural and urban residents. This suggests that the trend of perceived quality of primary care among rural and urban residents had the same variation trends before the HMS, confirming the common trend hypothesis.

### DID analysis of the impact of HMS on the perceived quality of primary care

Table 2 reports the estimated treatment effects of HMS implementation on the residents’ perceived quality of primary care. In model 1 without adjustment for control variables, the HMS implementation reduce the perceived quality of primary care among rural residents (OR: 0.79, 95%CI 0.67–0.95). After controlling the impacts of covariates in model 2, the negative impact of HMS implementation was found to decrease slightly (OR: 0.82, 95%CI 0.68–0.99). In contrast, although we found that the implementation of the HMS has been shown to increase the quality of primary care perceived by urban residents (OR: 1.13, 95%CI 0.87–1.46, model 2), such association failed to achieve significance in our study. There was a significant relationship between good self-reported health and patients’ perceived quality of primary care. There was no significant relationship between basic health insurance and the perceived quality of primary care.

**Table 2:** The impact of HMS on the perceived quality of primary care

	Rural		Urban	
	Model 1	Model 2	Model 1	Model 2
DID_HMS	0.79*** (0.67–0.95)	0.82** (0.68–0.99)	1.06 (0.83–1.36)	1.125 (0.87–1.46)
Male		0.47* (0.19–1.15)		1.907 (0.47–7.8)
Age		0.99 (0.94–1.04)		0.965 (0.82–1.14)
Married		1.2 (0.94–1.53)		0.888 (0.64–1.23)
Divorced or widowed		1.39* (0.99–1.95)		1.012 (0.65–1.57)
Primary school		1.26 (0.96–1.65)		1.47 (0.93–2.33)
Junior middle school and above		1.25 (0.87–1.78)		1.056 (0.6–1.85)
Economic level		1.03* (1–1.05)		1.02 (0.98–1.06)
Insured		1.03 (0.92–1.16)		0.924 (0.81–1.05)
Fair		0.98 (0.88–1.09)		0.952 (0.81–1.12)
Good		1.13** (1.02–1.25)		1.157* (0.99–1.36)
Very good		1.37*** (1.22–1.54)		1.294*** (1.08–1.55)
Excellent		1.75*** (1.54–1.99)		1.854*** (1.51–2.28)
Diagnosed with chronic diseases		1.10** (1.01–1.21)		1.092 (0.96–1.25)
Number of health workers in primary care facilities (per 1000 residents)		0.84 (0.61–1.14)		1.202 (0.78–1.84)
Year dummies	Yes	Yes	Yes	Yes
Individual effect	Yes	Yes	Yes	Yes
Controls	No	Yes	No	Yes
Observations	40 011	40 011	23 958	23 958

DID_HMS, the interactive term of whether or not implement HMS and the treated time. Model 1, odds ratio without controls are reported. Model 2, adjusted odds ratio with controls are reported. 95% CIs are mentioned in parentheses. All models used two-way fixed effects (year effect and individual effect), therefore errors are clustered at the residents level. This table was created by the coauthors of this manuscript. *p < 0.01, ** *p* < 0.05, *** *p* < 0.01

Table 3 presents the impact of implementation time on the estimated policy treatment effects. We found that there was a 24% reduction in the perceived quality of primary care (OR: 0.76, 95%CI 0.61–0.96, model 2) one year after the introduction of HMS among rural residents, and there was no statistical significance after the policy was implemented for two years, implying that the HMS has a short-term negative impact on quality of care perceived by rural residents.

**Table 3:** Long-term impact of HMS on the perceived quality of primary care

Variables	Rural		Urban	
	Model 1	Model 2	Model 1	Model 2
Post-intervention 1	0.81** (0.67–0.98)	0.76** (0.61–0.96)	1.30 (0.33–3.14)	1.42 (0.32–6.14)
Post-intervention 2	0.87 (0.62–1.23)	0.79 (0.51–1.24)	1.10 (0.07–6.17)	1.34 (0.07–24.51)
Year dummies	Yes	Yes	Yes	Yes
Individual effect	Yes	Yes	Yes	Yes
Controls	No	Yes	No	No
Observations	40 011	40 011	23 958	23 958

Post-intervention 1 means the impact after treated one period (current year); Post-intervention 2 means the impact after treated two period (two year)

Model 1, odds ratio without controls are reported. Model 2, adjusted odds ratio with controls are reported. 95% CIs are mentioned in parentheses. All models used two-way fixed effects (year effect and individual effect), therefore errors are clustered at the residents level. This table was created by the coauthors of this manuscript. **p < 0.05

### Analysis of the marginal impact of HMS on the perceived quality of primary care

Table 4 shows the marginal impact of HMS on the perceived quality of primary care in rural areas. After the implementation of the HMS, rural residents were 1%, 2%, and 3% more likely to perceive “very bad medical quality”, “bad medical quality”, and “average medical quality” of primary care, respectively, and their likelihood of perceiving “good medical quality” and “very good medical quality” of primary care decreased by 3% and 2%, respectively. We found that HMS had only a short-term impact on the perceived quality of primary care in rural areas and had no statistically significant impact on the quality of primary care perceived by urban residents (see Supplementary Table 5).

**Table 4:** Marginal impact of HMS on the perceived quality of primary care in rural area

Variables	Perceived quality of primary care				
	Very bad medical quality	Bad medical quality	Average medical quality	Good medical quality	Very good medical quality
DID_HMS	0.01** (0.01)	0.02** (0.01)	0.03** (0.01)	−0.03** (0.01)	−0.02** (0.01)
Post-intervention 1	0.01** (0.01)	0.02** (0.01)	0.03** (0.01)	−0.03** (0.01)	−0.02** (0.01)
Post-intervention 2	0.01 (0.01)	0.01 (0.01)	0.02 (0.01)	−0.02 (0.01)	−0.01 (0.01)
Year dummies	Yes	Yes	Yes	Yes	Yes
Individual effect	Yes	Yes	Yes	Yes	Yes
Controls	Yes	Yes	Yes	No	Yes
Observations	40 011	40 011	40 011	40 011	40 011

^**^p < 0.05

### Robustness checks of the impact of HMS on the perceived quality of primary care

The implementation of HMS had a negative impact on the perceived quality of primary care of rural residents when we used OLS estimation, which confirms the robustness of the main analysis (see Supplementary Table 6). The impact of HMS on individuals’ satisfaction is shown in Table 5. We found that rural residents’ satisfaction with primary care declined by 23% after HMS, while there is no statistical significance among urban residents. The results are consistent with those in Table 2, indicating that the results of the main analysis were robust and reliable. After advancing the implementation time of the policy, the HMS had no statistically significant effect on the quality of primary care perceived by rural or urban residents, which verifies the robustness of the DID analysis (see Supplementary Table 7).

**Table 5:** Robustness check-The impact of HMS on patient satisfaction of primary care facilities

	Rural		Urban	
	Model 1	Model 2	Model 1	Model 2
DID_HMS	0.77*** (0.65–0.92)	0.77*** (0.64–0.92)	1.12 (0.87–1.44)	1.12 (0.87–1.44)
Male		1.003 (0.37–2.71)		0.89 (0.23–3.49)
age		1.039 (0.96–1.13)		1.02 (0.86–1.21)
Married		1.13 (0.87–1.46)		1.23 (0.91–1.67)
Divorced or widowed		1.15 (0.82–1.64)		1.17 (0.74–1.84)
Primary school		0.85 (0.63–1.14)		1. (0.93–2.45)
Junior middle school and above		0.95 (0.66–1.36)		1.59 (0.9–2.84)
Economic level		1.03* (1–1.05)		1.01 (0.98–1.05)
Insured		0.95 (0.84–1.07)		0.99 (0.87–1.13)
Fair		0.96 (0.85–1.07)		0.96 (0.82–1.14)
Good		1.14** (1.03–1.26)		1.17* (1–1.37)
Very good		1.39*** (1.24–1.57)		1.33*** (1.11–1.6)
Excellent		1.711*** (1.5–1.95)		1.79*** (1.46–2.2)
Diagnosed with chronic diseases		1.06 (0.97–1.17)		1.13* (0.99–1.29)
Number of health workers in primary medical institutions per 1000 residents		0.89 (0.66–1.23)		1.28 (0.84–1.96)
Year dummies	Yes	Yes	Yes	Yes
Individual effect	Yes	Yes	Yes	Yes
Controls	No	Yes	No	Yes
Observations	40 011	40 011	23 958	23 958

^*^p < 0.01, **p < 0.05, ***p < 0.01

### Heterogeneity test of the impact of HMS on the perceived quality of primary care

Table 6 reports the heterogeneity test results for residents with and without chronic diseases. After HMS, the level of perceived quality of primary care by rural patients with chronic diseases decreased by 72%, whereas there was no statistically significant change in the perceived quality of primary care for urban residents with or without chronic diseases. After the implementation of HMS, the perceived quality of primary care by rural and urban patients over 60 years old decreased by 35% and 18%, respectively. HMS increased the quality of perceived primary care by 32% in urban patients under 45 years old. (see Supplementary Table 8).

**Table 6:** Heterogeneity test-Whether or not diagnosed with chronic diseases

Variables	Rural		Urban	
	Diagnosed with chronic diseases	No chronic diseases	Diagnosed with chronic diseases	No chronic diseases
DID_HMS	0.28** (0.11–0.78)	0.88 (0.71–1.08)	0.47 (0.16–1.38)	1.23 (0.92–1.65)
Year dummies	Yes	Yes	Yes	Yes
Individual effect	Yes	Yes	Yes	Yes
Controls	No	Yes	No	Yes
Observations	34 619	5 384	21 022	2 933

^**^p < 0.05

## Discussion

This study verified that HMS implementation had different impacts on the perceived quality of primary care among rural and urban residents. The perceived quality of primary care of rural residents decreased after HMS, whereas there was no significant change for urban residents. Furthermore, the long-term impact of HMS on the perceived quality of primary care was not statistically significant. Residents over 60 years old and rural residents with chronic diseases perceived poorer quality of primary care after the implementation of HMS.

We found that the implementation of the HMS resulted in an average decrease of 18% in the quality of primary care perceived among rural residents, which is similar to the results of the impact of hospital-township health centre integration [22]. A potential reason is the unbalanced distribution of healthcare resources, which are relatively scarce in rural areas [25]. Many studies have shown that the quality of care in primary care facilities in rural areas remains low due to a lack of health workforce, these providers cannot efficiently serve rural patients [26–28]. Secondly, the initial phase of the HMS focused on the overall improvement of the comprehensive service capacity of county hospitals, and most funding went to tertiary hospitals in urban areas and county hospitals [29]. A study from the same period found that the utilization of outpatient services at county hospitals increased by 56% and that the utilization at village clinics decreased by 44% in rural China [30]. The quality of primary care facilities is relatively low and difficult to improve in the short term. Therefore, the quality of medical services perceived by patients did not improve.

We found that the negative impacts of HMS among rural residents lasted only for a short period. It might be due to the following reasons: firstly, health practitioners and rural residents may have insufficient knowledge about the contents and benefits of the HMS, which leads to misunderstandings and dissatisfaction of residents during the process of medical care, and results in a decline in the perceived quality of primary care [31]. Secondly, the implementation of HMS requires a period of adaptation, and primary care facilities may face problems such as unstable service quality and insufficient allocation of healthcare resources, which results in a decrease in the perceived quality of rural residents in the short term. However, over time, some of these problems may be resolved, making the negative impact no longer significant.

We also found that the HMS improved the perceived quality of primary care among urban residents, but this association was not statistically significant. Consistent with other studies, a Northern California study demonstrated that lean primary care redesigns improved patient experiences of care, and a study in Quebec found the positive impact of introducing a family medicine group on patients’ perceived quality of relational and informational continuity of care [14, 15]. Existing literature compared the perceived quality of primary care in rural and urban areas, and they found that total scores of perceived quality of primary care in urban areas were lower than those in rural areas [32]. This may be explained by the condition that the implementation of the HMS contributed to an increasing number of primary care facilities in urban areas [31, 33], and the quality of primary care in terms of human resources and patient experience in urban areas has indeed improved after HMS [34]. More use of primary care services was associated with higher perceived quality of primary care [35, 36]. Zhou et al. found that urban residents were 26.1% more likely to visit primary care facilities after the implementation of HMS [37]. Therefore, HMS may improve the perceived quality of primary care services for urban residents by increasing their utilization of primary care services.

We did not find a significant relationship between basic health insurance and the perceived quality of primary care, which is similar to the results conducted in Ghana’s hospitals [20]. Similar to prior studies, patients who were divorced or widowed were found to have better-perceived quality of care in rural areas [13]. Consistent with prior studies, [36, 38] the results demonstrated that patients with chronic diseases perceived higher quality of primary care than those without. A study conducted in China found that tiered healthcare system reform was associated with better quality of medical services among urban chronic disease patients [39]. Contrasting to the above studies, we found that the implementation of HMS decreased the quality of primary care perceived by rural patients with chronic diseases and residents over 60 years old. This can be explained by the fact that the public health service system in rural areas is not yet perfect, and the training in chronic disease management strategies is not well implemented [40]. This prevents patients with chronic diseases from obtaining comprehensive and systematic health management services. Consequently, they have poor perceptions of the quality of medical services.

Our study has several limitations. First, Previous studies have mostly used the Primary Care Assessment Tool (PCAT) to assess patients’ perceived quality of healthcare services with different aspects in primary care, whereas we used the overall perceived quality of primary care, which cannot reflect the quality of numerous aspects. Second, the HMS was fully implemented in 2017, and the short observation period after the policy implementation made it difficult to determine the long-term impact of the policy. Third, the selection criterion that we only chose residents who usually go to primary care facilities may have caused potential selection bias. Fourth, this study was performed based on the Chinese population, and the limited generalizability of the observed results cannot be ignored. Finally, patient-perceived quality of primary care is influenced by both supply and demand sides, but we only control for one control variable on the supply side.

## Conclusions

The implementation of HMS had different impacts on patients’ perceived quality of primary care in rural and urban areas. The quality of primary care perceived by rural residents decreased after the HMS, while there was no change in the quality of care perceived by urban residents. The negative impact of HMS on the perceived quality of primary care was more pronounced among rural patients with chronic disease. Policy decision-makers are suggested to establish a national quality monitoring system through a strong collaboration among government, patients, and caregivers that incorporates patient experience as an essential standard to systematically evaluate the impacts of the reform. In addition, more efforts should be made to help vulnerable groups over 60 years old and rural patients with chronic diseases. Specifically, one-stop outpatient clinics for chronic diseases can be set up to simplify the process of medical treatment; and conduct regular home follow-up visits for vulnerable groups.

## Supplementary Information

Additional file 1.

## Data Availability

The data used in this study are available on the CFPS website at http://www.isss.pku.edu.cn/cfps/index.htm.
