# Supplementary material for: Impact of the hierarchical medical system on the perceived quality of primary care in China: a quasi-experimental study
Source: Glob Health Res Policy. 2025 Feb 19;10:5. doi: 10.1186/s41256-024-00398-3 (PMC11837713; doi:10.1186/s41256-024-00398-3)
Supplement: Supplementary file 1 — Additional file 1. [file 41256_2024_398_MOESM1_ESM.docx]

**Impact of the hierarchical medical system on the perceived quality of primary care in China:**

**A quasi-experimental study**

**(****Supplementary Tables)**

**Contents**

[**Supplementary Table 1-The implementation of hierarchical medical system in China** 1](#_Toc181370519)

[**Supplementary Table 2-The sample of various places at different waves and phases** 3](#_Toc181370520)

[**Supplementary Table 3-Variable definitions** 3](#_Toc181370521)

[**Supplementary Table 4-Questions and answers of outcome variables in CFPS** 4](#_Toc181370522)

[**Supplementary Table 5-Marginal impact of HMS on perceived quality of primary care in urban area** 5](#_Toc181370523)

[**Supplementary Table 6-Robustness check-The impact of HMS using OLS regression** 5](#_Toc181370524)

[**Supplementary Table 7-Robustness check-advance treated time** 6](#_Toc181370525)

[**Supplementary Table 8- Heterogeneity test-different types of age** 7](#_Toc181370526)

**Supplementary Table 1-The implementation of hierarchical medical system in China**

| Province | Time for pilot implementation | Time for full implementation |
| --- | --- | --- |
| Beijing | / | “Key Tasks for the Construction of the Hierarchical Medical System in Beijing in 2016-2017”-Second half of 2016 |
| Tianjin | 4 districts started up before the end of 2015;  Hedong district, Nankai district and Hebei district implemented at the beginning of 2016 | 2016 |
| Shanghai | Some districts piloted in 2015 | June 2015; family doctor contract services |
| Zhejiang | Yiwu city, Shaoxing city and Wenzhou city, and 8 counties such as Chunan county implemented before the end of 2014;  16 counties and districts such as Yuhuan county implemented in March 2015 | “Implementation Opinions of the General Office of the People's Government of Zhejiang Province on Promoting the Construction of a Hierarchical Medical System”-June 2016 |
| Jiangsu | Every city chose 1 district and 1 county piloted in 2015 | “The construction of the hierarchical medical system of Jiangsu Province”-August 2015 |
| Guangdong | Zhuhai city implemented in May 2016 | "Implementation Plan for Accelerating the Construction of a Hierarchical Medical System in Guangdong Province"-2016 |
| Fujian | Putian city implemented in September 2015 | 2016 |
| Heilongjiang | Rural area piloted in September 2014 | October 2015 |
| Liaoning | Benxi city and Anshan city implemented before the end of 2015; | The General Office of the People's Government of Liaoning Province on promoting  Implementation Opinions on the Construction of Hierarchical Diagnosis and Treatment System-the end of 2015 |
| Jilin | Changchun city implemented in December 2016 | “Implementation Plan for Promoting the Construction of a Hierarchical Medical System in Jilin Province”-Second half of 2016 |
| Shandong | Weihai city, Dongying city and Weifang city, and 22 counties implemented in 2015 | Implementation Opinions on Promoting the Construction of Hierarchical Medical System"-Second half of 2015 |
| Shanxi | 18 counties implemented in September 2014 | "Implementation Opinions of the General Office of the People's Government of Shanxi Province on Establishing a Hierarchical Medical System"-Before the end of 2015 |
| Hubei | 28 counties and districts such as Yichang city and Xiangyang city implemented in October 2014 | “Implementation Plan for Promoting the Construction of Hierarchical Medical System in Hubei Province”-February 2016 |
| Jiangxi | Jingdezhen city and Jiujiang city started in September 2016 | Second half of 2016 |
| Hunan | Changsha city and Zhuzhou city implemented in July 2016 | “Implementation Opinions of the General Office of the People's Government of Hunan Province on Promoting the Construction of Hierarchical Medical System”-Second half of 2016 |
| Hebei | Tangshan city and Handan city implemented in 2015 | “Implementation Opinions on the Construction of Hierarchical Medical System in Hebei Province”-2016 |
| Henan | Zhengzhou city, Jiaozuo city, Luoyang city, puyang city, Mengjin county and Xinan county implemented in April 2016 | “Implementation Opinions on the Construction of Hierarchical Medical System in Henan Province”-2016 |
| Anhui | Bengbu city, Xuancheng city implemented in 2015 | “Implementation Opinions on the Construction of Hierarchical Medical System in Henan Province”-2016 |
| Chongqing | Yongchuan district, Jiulongpo district implemented in 2015 | 2016 |
| Yunnan | Yuxi city implemented in April 2016 | "Implementation Opinions of the General Office of the People's Government of Yunnan Province on Establishing and Improving the Hierarchical Diagnosis and Treatment System"-2016 |
| Shaanxi | 1. Baoji City and 10 counties implemented in July 2015;  2. Guanzhong area were implemented in October 2015 | “Implementation Opinions on the Construction of Hierarchical Medical System in Shaanxi Province”-Before the end of 2015 |
| Sichuan | / | “Implementation Opinions on the Construction of Hierarchical Medical System in Sichuan Province”-October 2014 |
| Gansu | November 2014 | “Implementation Opinions on the Construction of Hierarchical Medical System in Sichuan Province”-2016 |
| Guizhou | / | 2017 |
| Guangxi | Liuzhou city and Yulin city implemented in 2015 | “Implementation Opinions on Promoting the Construction of Hierarchical Medical System in the General Office of the People's Government of Guangxi Zhuang Autonomous Region ”-2016 |

Data resource: The official website of the Health Commission and the government of each province in China. / not applicable.

**Supplementary Table 2-The sample of various places at different waves and phases**

| Province | 2012 | 2014 | 2016 | 2018 | Pre-intervention | Pre-intervention |
| --- | --- | --- | --- | --- | --- | --- |
| Beijing | 14 | 30 | 57 | 63 | 101 | 63 |
| Tianjin | 62 | 66 | 60 | 64 | 128 | 124 |
| Shanghai | 568 | 581 | 496 | 353 | 1149 | 849 |
| Zhejiang | 237 | 289 | 264 | 254 | 526 | 518 |
| Jiangsu | 372 | 375 | 368 | 267 | 747 | 635 |
| Guangdong | 1539 | 1660 | 1592 | 1393 | 3199 | 2985 |
| Fujian | 228 | 237 | 224 | 222 | 465 | 446 |
| Heilongjiang | 233 | 235 | 205 | 168 | 468 | 373 |
| Liaoning | 1217 | 1238 | 1147 | 977 | 2455 | 2124 |
| Jilin | 198 | 200 | 217 | 175 | 615 | 175 |
| Shandong | 952 | 949 | 921 | 802 | 1901 | 1723 |
| Shanxi | 723 | 772 | 779 | 654 | 1495 | 1433 |
| Hubei | 221 | 199 | 199 | 184 | 420 | 383 |
| Jiangxi | 358 | 334 | 349 | 305 | 1041 | 305 |
| Hunan | 305 | 353 | 355 | 318 | 1013 | 318 |
| Hebei | 1218 | 1223 | 1191 | 1105 | 2441 | 2296 |
| Henan | 2481 | 2633 | 2473 | 2101 | 5114 | 4574 |
| Anhui | 313 | 335 | 294 | 269 | 648 | 563 |
| Chongqing | 149 | 123 | 146 | 136 | 272 | 282 |
| Yunnan | 599 | 693 | 612 | 612 | 1292 | 1224 |
| Shaanxi | 312 | 320 | 340 | 289 | 632 | 629 |
| Sichuan | 884 | 913 | 1002 | 885 | 1797 | 1887 |
| Gansu | 2330 | 2361 | 2323 | 1999 | 4691 | 4322 |
| Guizhou | 410 | 389 | 449 | 323 | 1248 | 323 |
| Guangxi | 350 | 403 | 443 | 361 | 753 | 804 |

**Supplementary Table 3-Variable definitions**

| Variables | Types of variables | Description |
| --- | --- | --- |
| Dependent variables |  |  |
| Perceived quality of primary care | Categorical variable | 1=Very bad medical quality, reference |
|  |  | 2=bad medical quality |
|  |  | 3=Average medical quality |
|  |  | 4=Good medical quality |
|  |  | 5=Very good medical quality |
| Patient satisfaction of primary care facilities ^a^ | Categorical variable | 1=Very dissatisfied, reference |
|  |  | 2=Dissatisfied |
|  |  | 3=Average |
|  |  | 4=Satisfied |
|  |  | 5=Very satisfied |
| Independent variables |  |  |
| Treat | Binomial variable | 0 = Control group  1 = Treatment group |
| Time | Binomial variable | 0 = Pre-intervention  1 = Post-intervention |
| Control variables |  |  |
| Gender | Categorical variable | 0=Female, 1=Male |
| Age (years) | Continuous variable | Continuous variable |
| Marital status | Categorical variable | 1=Unmarried, reference |
|  |  | 2=Married |
|  |  | 3=Divorced or widowed |
| Education status | Categorical variable | 1=Illiterate, reference |
|  |  | 2=Primary school |
|  |  | 3=Junior middle school and above |
| Household income per capita (CNY) | Continuous variable | Continuous variable |
| Basic health insurance ^b^ | Binomial variable | 0=Uninsured, 1=Insured |
| Self-reported health (SRH) | Categorical variable | 1= Poor, reference |
|  |  | 2= Fair |
|  |  | 3=Good |
|  |  | 4=Very good |
|  |  | 5=Excellent |
| Chronic diseases | Binomial variable | 0=No, 1=Yes |
| Number of health workforce in primary medical institutions per 1000 residents | Continuous variable | Continuous variable |

^a^ means the variable isn’t main dependent variable, it only used in robustness check.

**Supplementary Table 4-Questions and answers of outcome variables in CFPS**

| Question | Answers |
| --- | --- |
| 1. Which medical institutions you usually visit if you are sick or need advice about your health? | 1=General hospitals  2=Specialized hospitals  3=Community health centers/township health centers  4=Community health stations/village clinics 5=Private clinics |
| 2. What do you think about the medical quality of the medical institutions you usually visit? | 1=Very bad medical quality  2=Bad medical quality  3=Average medical quality  4=Good medical quality  5=Very good medical quality |
| 3. Are you satisfied with the overall condition of the medical institutions you usually visit? | 1= Very dissatisfied  2= Dissatisfied  3= Average  4= Satisfied  5= Very satisfied |

**Supplementary Table 5-Marginal impact of HMS on perceived quality of primary care in urban area**

| Variables | Perceived quality of primary care | | | | |
| --- | --- | --- | --- | --- | --- |
|  | Very bad medical quality | Bad medical quality | Average medical quality | Good medical quality | Very good medical quality |
| DID_HMS | 0.01 (0.01) | -0.01 (0.01) | -0.02 (0.01) | 0.02 (0.01) | 0.01 (0.01) |
| Post-intervention 1 | 0.01 (0.01) | 0.02 (0.01) | 0.03 (0.01) | -0.03 (0.01) | -0.02 (0.01) |
| Post-intervention 2 | 0.01 (0.01) | 0.01 (0.01) | 0.02 (0.01) | -0.02 (0.01) | -0.01 (0.01) |
| Year dummies | Yes | Yes | Yes | Yes | Yes |
| Individual effect | Yes | Yes | Yes | Yes | Yes |
| Controls | Yes | Yes | Yes | No | Yes |
| Observations | 40 011 | 40 011 | 40 011 | 40 011 | 40 011 |

**Supplementary Table 6-Robustness check-The impact of HMS using OLS regression**

|  | Rural | |  | Urban | |
| --- | --- | --- | --- | --- | --- |
|  | Model 1 | Model 2 |  | Model 1 | Model 2 |
| DID_HMS | -0.08** | -0.06** |  | 0.02 | 0.04 |
|  | (0.03) | (0.03) |  | (0.04) | (0.04) |
| Gender |  | -0.25* |  |  | 0.23 |
|  |  | (0.14) |  |  | (0.24) |
| Age |  | -0.01 |  |  | -0.01 |
|  |  | (0.01) |  |  | (0.02) |
| Married |  | 0.06 |  |  | -0.04 |
|  |  | (0.04) |  |  | (0.05) |
| Divorced or widowed |  | 0.11* |  |  | 0.02 |
|  |  | (0.06) |  |  | (0.07) |
| Primary school |  | 0.08 |  |  | 0.15* |
|  |  | (0.05) |  |  | (0.08) |
| Junior middle school and above |  | 0.07 |  |  | 0.05 |
|  |  | (0.06) |  |  | (0.10) |
| Household income per capita |  | 0.01* |  |  | 0.01 |
|  |  | (0.01) |  |  | (0.01) |
| Insured |  | 0.01 |  |  | -0.02 |
|  |  | (0.02) |  |  | (0.02) |
| Fair |  | -0.01 |  |  | -0.01 |
|  |  | (0.02) |  |  | (0.03) |
| Good |  | 0.04** |  |  | 0.05* |
|  |  | (0.02) |  |  | (0.02) |
| Very good |  | 0.10*** |  |  | 0.08*** |
|  |  | (0.02) |  |  | (0.03) |
| Excellent |  | 0.18*** |  |  | 0.19*** |
|  |  | (0.02) |  |  | (0.03) |
| Diagnosed with chronic diseases |  | 0.03** |  |  | 0.03 |
|  |  | (0.02) |  |  | (0.02) |
| Number of health workforce in primary medical institutions per 1000 residents |  | -0.06  (0.05) |  |  | 0.06  (0.06) |
| Constant | 3.34*** | 3.49*** |  | 3.29*** | 3.45*** |
|  | (0.01) | (0.39) |  | (0.01) | (0.95) |
| Year dummies | Yes | Yes |  | Yes | Yes |
| Individual effect | Yes | Yes |  | Yes | Yes |
| Controls | No | Yes |  | No | Yes |
| Observations | 40 011 | 40 011 |  | 23 958 | 23 958 |

Model 1, coefficient without controls are reported. Model 2, adjusted coefficient with controls are reported. Std.Error are mentioned in parentheses. All models used two-way fixed effects (year effect and individual effect), therefore errors are clustered at the residents level. This table was created by the coauthors of this manuscript. * *p*<0.01, ** *p*<0.05, *** *p*<0.01.

**Supplementary Table 7-Robustness check-advance treated time**

| Variables | Rural | |
| --- | --- | --- |
|  | Model 1 | Model 2 |
| **Advance 1 period** | 1.10 (0.91 to 1.32) | 1.09 (0.89 to 1.33) |
| Advance 2 period | 1.03 (0.86 to 1.22) | 1.01 (0.84 to 1.22) |
| Year dummies | Yes | Yes |
| Individual effect | Yes | Yes |
| Controls | No | Yes |
| Observations | 40 011 | 40 011 |

Because we didn’t find the significant impact of HMS on perceived quality of primary care in urban area, we didn’t report the results of advance treated time.

**Supplementary Table 8- Heterogeneity test-different types of age**

| Variables | Rural |  |  |  | Urban | | |
| --- | --- | --- | --- | --- | --- | --- | --- |
|  | <45 | 45-59 | >60 |  | <45 | 45-59 | >60 |
| DID_HMS | 0.68** (0.47 to 0.99) | 0.84 (0.58 to 1.22) | 0.65** (0.45 to 0.92) |  | 1.32* (0.85 to 2.05) | 1.42 (0.83 to 2.41) | 0.82** (0.44 to 1.52) |
| Year dummies | Yes | Yes | Yes |  | Yes | Yes | Yes |
| Individual effect | Yes | Yes | Yes |  | Yes | Yes | Yes |
| Controls | Yes | Yes | Yes |  | Yes | Yes | Yes |
| Observations | 16 026 | 13 657 | 10 328 |  | 11 078 | 7 344 | 5 536 |

* *p*<0.01, ** *p*<0.05.
